# Transcriptome Analysis Reveals that Red and Blue Light Regulate Growth and Phytohormone Metabolism in Norway Spruce [*Picea abies* (L.) Karst.]

**DOI:** 10.1371/journal.pone.0127896

**Published:** 2015-08-03

**Authors:** Fangqun OuYang, Jian-Feng Mao, Junhui Wang, Shougong Zhang, Yue Li

**Affiliations:** 1 State Key Laboratory of Tree Genetics and Breeding, Research Institute of Forestry, Chinese Academy Forestry, Beijing, 100091, PR China; 2 National Engineering laboratory for Forest Tree Breeding, Key Laboratory for Genetics and Breeding of Forest Trees and Ornamental Plant of Ministry of Education, College of Biological Science and Technology, Beijing Forestry University, Beijing, 100083, PR China; Henan Agricultural Univerisity, CHINA

## Abstract

The mechanisms by which different light spectra regulate plant shoot elongation vary, and phytohormones respond differently to such spectrum-associated regulatory effects. Light supplementation can effectively control seedling growth in Norway spruce. However, knowledge of the effective spectrum for promoting growth and phytohormone metabolism in this species is lacking. In this study, 3-year-old Norway spruce clones were illuminated for 12 h after sunset under blue or red light-emitting diode (LED) light for 90 d, and stem increments and other growth traits were determined. Endogenous hormone levels and transcriptome differences in the current needles were assessed to identify genes related to the red and blue light regulatory responses. The results showed that the stem increment and gibberellin (GA) levels of the seedlings illuminated by red light were 8.6% and 29.0% higher, respectively, than those of the seedlings illuminated by blue light. The indoleacetic acid (IAA) level of the seedlings illuminated by red light was 54.6% lower than that of the seedlings illuminated by blue light, and there were no significant differences in abscisic acid (ABA) or zeatin riboside [ZR] between the two groups of seedlings. The transcriptome results revealed 58,736,166 and 60,555,192 clean reads for the blue-light- and red-light-illuminated samples, respectively. Illumina sequencing revealed 21,923 unigenes, and 2744 (approximately 93.8%) out of 2926 differentially expressed genes (DEGs) were found to be upregulated under blue light. The main KEGG classifications of the DEGs were metabolic pathway (29%), biosynthesis of secondary metabolites (20.49%) and hormone signal transduction (8.39%). With regard to hormone signal transduction, *AUXIN-RESISTANT1* (*AUX1*), *AUX/IAA* genes, auxin-inducible genes, and early auxin-responsive genes [(*auxin response factor* (*ARF*) and *small auxin-up RNA* (*SAUR*)] were all upregulated under blue light compared with red light, which might have yielded the higher IAA level. *DELLA* and phytochrome-interacting factor 3 (PIF3), involved in negative GA signaling, were also upregulated under blue light, which may be related to the lower GA level. Light quality also affects endogenous hormones by influencing secondary metabolism. Blue light promoted phenylpropanoid biosynthesis, phenylalanine metabolism, flavonoid biosynthesis and flavone and flavonol biosynthesis, accompanied by upregulation of most of the genes in their pathways. In conclusion, red light may promote stem growth by regulating biosynthesis of GAs, and blue light may promote flavonoid, lignin, phenylpropanoid and some hormones (such as jasmonic acid) which were related to plant defense in Norway spruce, which might reduce the primary metabolites available for plant growth.

## Introduction

Light quality [1–3] has important effects on plant growth and development, especially for plants in high-latitude areas [4], and different light spectra have different effects on plant growth [5]. Studies to date of the effects of light quality have mainly concentrated on model plants [6, 7], algae [8, 9], and vegetables [10–12]. By contrast, there are few studies of the effects of light quality on woody plants. Thus, it is of great importance to increase the current understanding of the growth response of woody plants to light quality.

The spectra of sunlight that affect plant photosynthesis primarily include red and blue light. Blue light, which has a shorter wavelength and higher energy than red light, has been found to promote hydraulic conductivity in *Betula pendula* [13]. However, blue light does not have a significant effect on hypocotyl extension in Scots pine (*Pinus sylvestris* L.), a species in which stem extension is regulated by far-red light [14]. Mølmann et al. (2006) [15] have found that red and far-red light can maintain the growth of Norway spruce and that a southern population is more sensitive to red light, lacking a complete bud set, even at a low level of radiation (0.1 Wm^-2^). However, blue light induces bud set in seedlings. Furthermore, the effects of light quality vary among different varieties or species of plants. The different mechanisms by which light quality regulates plant growth and development include the selective activation of all types of light receptors, such as the activation of phytochrome by red and far-red light, cryptochrome and phototropin by blue light, and UVB receptor by ultraviolet light [3, 16].

Plant growth is also affected by interactions between endogenous hormone levels and light quality [17]. In the light regulation process, the hormone level in a plant affects its light responsiveness. Exogenous hormones can stimulate the light-mediated regulation of plant growth, functioning as second messengers in light signal transduction processes [18]. In turn, light regulates a variety of hormone pathways. *PHYA* affects the hybrid aspen gibberellin (GA) and indoleacetic acid (IAA) metabolic pathways [19], and key light signaling components, such as phytochrome-interacting factor 3 (*PIF3)*, *PIF4* and *HY5*, can connect light and plant hormone signaling in the regulation of seedling photomorphogenesis [17]. The plant hormones associated with light-mediated plant growth regulation largely include GAs [6, 20, 21], auxins [22, 23], cytokinins [20] and abscisic acid (ABA) [24], of which the growth-promoting phytohormones GAs and auxin play the main roles. Light quality also affects endogenous hormone regulation of plant growth and development by influencing secondary metabolism; for example, blue light promotes flavonoid accumulation [25], which affects auxin polar transport [26].

Light supplementation can effectively control seedling growth in Norway spruce. However, knowledge of the effective spectrum for promoting growth and phytohormone metabolism in this tree is still lacking. The completion of the *P*. *abies* genome [27] and the rapid development of high-throughput sequencing have facilitated gene expression studies in Norway spruce using RNA sequencing (RNA-seq) analysis. In the present study, 3-year-old *P*. *abies* clones were illuminated for 12 h after sunset under red or blue LED light, and stem increment and other growth trait determinations, phytohormone level measurements and RNA-seq analysis were performed to achieve the following aims: (1) to understand the effects of these two types of light qualities on Norway spruce growth; (2) to analyze the relationship between light quality and plant hormones in Norway spruce; and to identify differentially expressed genes (DEGs) under red and blue light. This study was conducted to provide a basis for elucidating the genetic mechanisms by which different light qualities regulate seedling growth and phytohormone levels.

## Materials and Methods

### Experimental design and growth conditions

*P*. *abies* clones (3 years old) were grown in a container (10 x 10 cm) under greenhouse conditions and received natural light during the day and illumination by blue-light (B, 460 nm) or red-light (R, 660 nm) LED lamps for 12 h at night (power of 90 W) from May 10 to August 10, 2013. Each LED lamp was 90 cm long and was installed above five clones. Each row included five clones arranged with 10 cm spacing. Light intensity (50 μmol.m^-2^.s^-1^) was measured using a spectrum radiator (OL750) at the National Institute of Metrology, People’s Republic of China. Five clones were present in each plot, with three plot replications per light quality treatment. Shading cloth was used between the plots during the lighting period and was removed during the day.

The clones were tended consistently for all experimental treatments. From June until September, the average temperature in the nursery was 20~26°C, with an average humidity of 50~65%. The clones were watered regularly and fertilized twice per month using a foliar nutrient with 5 kg/m^2^ 4/1000 monopotassium phosphate (the main ingredient was KH_2_PO_4_) and 0.002 kg/kg phosham [the main ingredient was (NH_4_)_2_HPO_4_], calcium phosphate [Ca_3_(PO4)_2_], and carbamide (H_2_NCONH_2_).

### Measurements of seedling traits

We determined the stem height, ground diameter, and length of the current shoots for all clones in August 2013 after 90 days of illumination. The leaf area and leaf weight were measured for 30 needles from 6 clones per LED treatment. The specific leaf weight (SLW) was calculated as follows: leaf weight/leaf area.

### Determination of plant hormones

In August 2013, after 90 days of blue or red light treatment, current needles were harvested from three randomly selected Norway spruce clones and stored at -80°C until analysis for the following phytohormones: GAs, IAA, ABA, and ZR. The samples were ground in an ice-cold mortar, extracted with 10 mL 80% methanol (v/v) containing 1 mmol^-1^ butylated hydroxytoluene (BHT), and then stored at 4°C for 4 h. Next, they were centrifuged at 3500 rpm for 8 min at 4°C. The precipitation was extracted with the same extraction solution for 1 h at 4°C and then centrifuged again under the same conditions. The two combined supernatants were passed through Chromosep C18 columns (C18 Sep-Park Cartridge, Waters Corp., Milford, MA) and dried under N_2_. The residues were then dissolved in 2 mL phosphate-buffered saline (PBS) for analysis of GAs, IAA, ABA, and ZR using an enzyme-linked immunosorbent assay (ELISA). Mouse monoclonal antigens and antibodies against IAA, GAs, ZR and ABA and IgG-horseradish peroxidase used in ELISA were obtained from the Research Institute of China Agricultural University (Beijing, China). Microtiter plates (96-well) were coated with 100 μL of coating buffer (1.5 g L^-1^ Na_2_CO_3_, 2.93 g L^-1^ NaHCO_3_, and 0.02 g L^-1^ NaN_3_, pH 9.6) containing 0.25μg mL^-1^ of antigens against the hormones and were incubated for 4 h at 37°C for ZR, GAs, and ABA and overnight at 4°C for IAA. Then, the plates were washed four times with PBS + Tween 20 (0.1% [v/v]) buffer (pH 7.4), coated with 50 L^-1^ of either grain extracts or hormone standards and 50 μL of 20μg mL^-1^ antibodies and incubated for 3 h at 28°C for ZR, GAs, and ABA and overnight at 4°C for IAA. After washing again as above, 100 μL of IgG-horseradish peroxidase substrate was added and incubated for 1 h at 30°C. A color development solution containing 1.5 mg mL^-1^ 0-phenylenediamine and 0.008% (v/v) H_2_O_2_ was added to each well of the plate, after the wells were washed five times with the above mentioned PBS + Tween 20 buffer. An ELISA reader (model EL310, Bio-TEK, Winooski, VT) was used to detect the color development in each well at optical density A490, after the reaction progress was stopped by adding 50 μL of 2 mol.L-1 H_2_SO_4_ per well. The ZR, IAA, GAs, and ABA concentrations were calculated according to Weiler et al. (1981)[28].

### RNA-seq analysis

#### RNA extraction, cDNA library preparation, and sequencing

Total RNA was extracted from 50 to 100 mg of needles using TRIZOL reagent (Invitrogen, Carlsbad, CA, USA) according to the manufacturer’s instructions. The samples were subjected to residual DNA removal by DNase I digestion for 30 min at 37°C (Takara, Dalian, China). Then, a NanoDrop ND-1000 spectrophotometer (LabTech, Holliston, MA, USA) was used to measure the absorbance of the purified RNA at 260 and 280 nm (A260/A280) to determine the quality and quantity. The average RNA integrity number (RIN) of the samples was 8.9 using an Agilent 2100 Bioanalyzer (Agilent Technologies, Santa Clara, CA, USA). Next, mRNA was purified from 6 μg of total RNA (an equal-ratio mixture of RNA from three randomly selected blue- and red-light-treated *P*. *abies* needles) using oligo (dT) magnetic beads to remove the rRNA. A fragmentation buffer was added to produce short mRNA fragments (approximately 200 bp long). First-strand cDNA was synthesized using random hexamer primers with mRNA fragments as templates. Second-strand cDNA was synthesized by adding buffer, dNTPs, RNase H, and DNA polymerase I. The double-stranded cDNA was purified using a QiaQuick PCR Extraction Kit and washed with EB buffer for end repair and single nucleotide A (adenine) addition. In the end, sequencing adaptors were ligated to the fragments. The required fragments were purified using agarose gel electrophoresis and enriched by PCR amplification. The library products were prepared for sequencing analysis using an Illumina HiSeq 2000 (Illumina, San Diego, CA, USA).

#### Sequence read mapping and assembly

Clean reads were generated by filtering raw reads, removing low-quality tags (reads with unknown nucleotides, “N”), removing empty reads (no read sequence between the adaptors), and removing reads with only one copy number (that may have resulted from sequencing errors). Reference genome and gene model annotation files were downloaded from the Norway spruce genome website at http://congenie.org/eplant. The remaining clean reads were aligned to the reference genome and sequences using SOAPaligner/soap2, permitting up to two base mismatches. The alignment data were used to calculate the distribution of reads for the reference genes and to perform coverage analysis.

#### Quantification and differential expression analysis of transcripts

The reads per kilobase transcriptome per million mapped reads (RPKM) method [29] was used to calculate the gene expression level following the formula: *RPKM* = 10^6^ *C*/(*NL*/10^3^). When calculating the expression of gene A, C represents the reads uniquely aligned to gene A, N is the total number of reads uniquely aligned to all genes, and L is the number of bases in gene A. We used “FDR ≤ 0.001 [7] and the absolute value of Log_2_Ratio ≥ 1” as thresholds to determine the significance of the gene expression differences, where FDR is the false discovery rate. More stringent criteria, including smaller FDRs and larger fold changes, can be used to identify DEGs.

#### Gene Ontology (GO) and Kyoto Encyclopedia of Genes and Genomes (KEGG) enrichment analysis of differentially expressed transcripts

GO and KEGG enrichment analyses were performed to assess the DEGs. GO terms with corrected P-values of less than 0.05 were considered to be significantly enriched in the DEG transcripts. For KEGG analysis, we used a Q-value of less than or equal to 0.05 as the threshold to demonstrate significant enrichment of the gene sets.

### Quantitative real-time PCR (qRT-PCR) validation

The DEGs with three biological replicates were validated using qRT-PCR. In all cases, the primers designed spanned exon-exon boundaries. Actin AAF03692 (TGAGCTCCCTGATGGGCAGGTGA/TGGATACCAGCAGCTTCCATCCCAAT) [30] was used as a reference control. The reaction was performed using a 26SYBR Green Master Mix (TianGen) and a CFX96 Real-Time System (Bio-Rad, USA) under the following conditions: denaturation at 95°C for 3 min followed by 40 cycles of amplification (95°C for 30 s, 60°C for 30 s, and 72°C for 30 s). Relative expression was calculated using the delta-delta-Ct method. The primer sequences can be found in S1 Table.

## Results

### Effects on seedling traits

**Fig 1:**
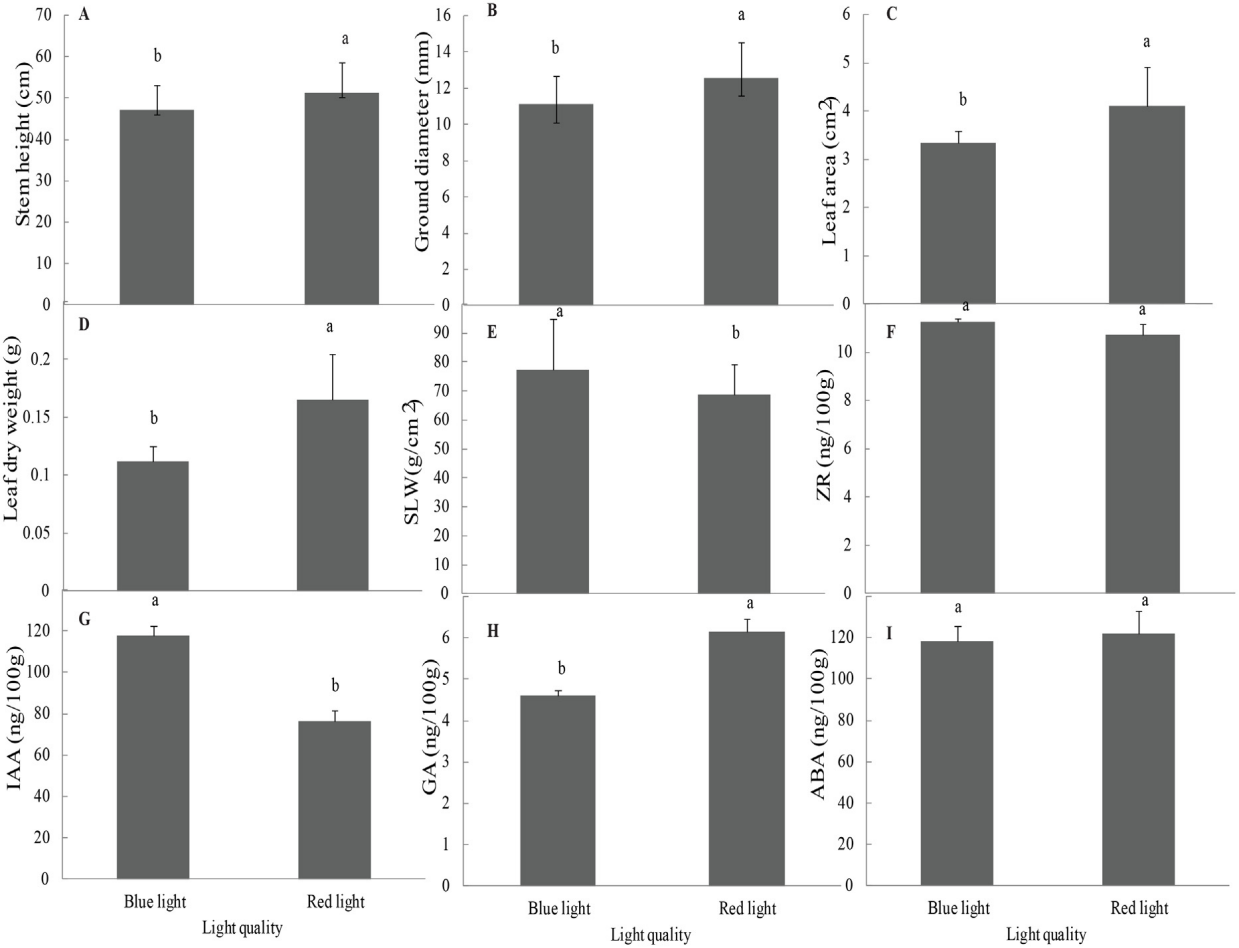
Multiple comparisons of growth traits and phytohormone average levels for red light and blue light treatments.

There were significant differences in stem height, ground diameter, leaf area, leaf dry weight, and SLW between the two light quality treatments (P-value < 0.1). Multiple comparisons of the data showed that the average values under the red light treatment were higher than those under the blue light treatment, except for SLW (Fig 1A–1E). The average stem increment, ground diameter, leaf area, and leaf dry weight of the clones illuminated by red light were 8.6%, 13.27%, 22.72%, and 47.32% higher, respectively, than those of the seedlings illuminated by blue light, whereas the average SLW was 11.44% lower than that in the seedlings illuminated by blue light.

### Effects on phytohormones

ZR and ABA were not affected by light quality, whereas it had significant effects on GAs and IAA (P-value < 0.1). The average GA level of the seedlings illuminated by red light (6.14 ng/100 g) was 29.0% higher than that of those illuminated by blue light (4.6 ng/100 g). Conversely, the IAA level (76.22 ng/100 g) was 54.6% lower compared with the seedlings illuminated by blue light (117.8 ng/100 g) (Fig 1H–1K).

### RNA-seq analysis

The focus of this study was to identify candidate genes for investigating light quality (red light and blue light) responses based on DEG patterns in Norway spruce clones grown under red and blue light. Therefore, we examined the changes in gene expression under red light using the expression levels under blue light as a reference, and *vice versa*. Using this approach, the genes enhanced under red light were found to be suppressed under blue light, and *vice versa*.

### Illumina sequencing and mapping of the reference genome and genes

**Fig 2:**
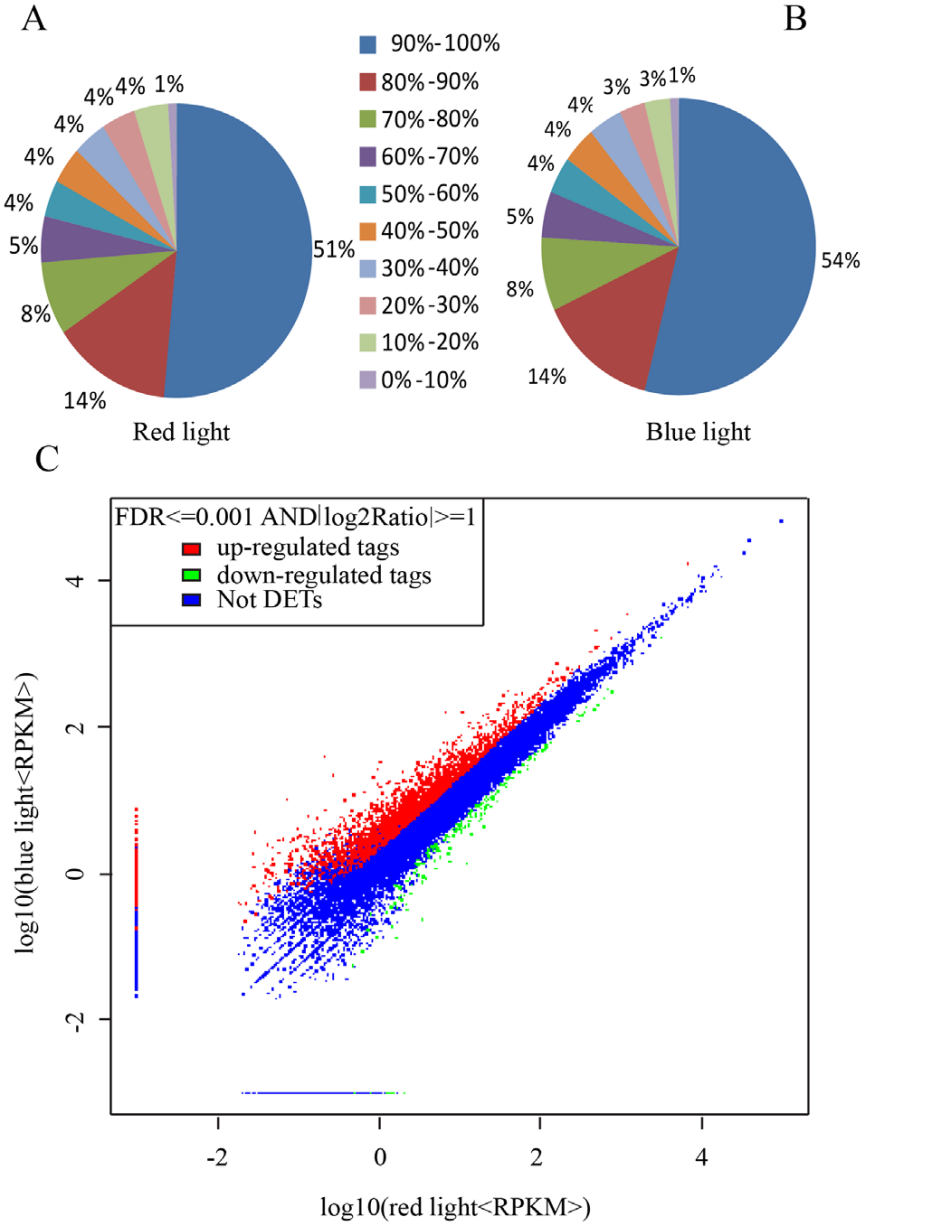
Mean coverages of all genes in the samples treated with red light (A) and blue light (B) and the upregulated and downregulated DEGs in the sample treated with blue light compared with that treated with red light (C).

In this study, 58,736,166 and 60,555,192 clean reads were generated for each sample illuminated by red or blue LED lamps, which were used for further analysis (Table 1). Approximately 71% of the reads in each sample were uniquely mapped to the Norway spruce genome, and 36% of the total reads were assigned to Norway spruce genes (Table 1). A total of 20,817 and 21,414 genes (short reads mapped to the reference genome) were detected in each library, accounting for more than 78% of the 26,437 genes in the *P*. *abies* reference genome database (Nystedt et al., 2013). Overall, 21,923 unique genes were expressed between the two libraries (S2 Table), with a mapping coverage of 82.93%, which strongly supported the RNA-seq results. The mean coverage of all genes was greater than 78%, and the highest coverage achieved was 100.00% (Fig 2A and 2B).

**Table 1:** Mapping of RNA-seq library reads in *Picea abies* under red light and blue light to the *Picea abies* reference genome and genes.

Classification	Red light				Blue light			
	Number	Map to genome (%)	Number	Map to gene (%)	Number	Map to genome (%)	Number	Map to gene (%)
**Raw reads**	60,555,192	100	60,555,192	100	58,736,166	100	58,736,166	100
**Base pairs**	5,449,967,280	100	5,449,967,280	100	5,286,254,940	100	5,286,254,940	100
**Mapped reads**	43,218,113	71.4	22,730,112	37.54	5,727,198	71.3	21,281,009	36.23
**Perfect match**	24,893,891	41.1	14,386,804	23.76	3,358,895	41.2	13,580,829	23.12
**< = 5 bp mismatch**	18,324,222	30.3	8,343,308	13.78	2,368,303	30.1	7,700,180	13.11
**Unique match**	38,131,112	63.0	22,097,891	36.49	5,510,915	62.5	20,714,206	35.27
**Multi-position match**	5,087,001	8.4	632,221	1.04	216,283	8.8	566,803	0.96
**Unmapped reads**	17,337,079	28.6	37,825,080	62.46	53,008,968	28.7	37,455,157	63.77

### DESeq analysis

In total, 20,308 uniform genes from the two libraries were analyzed using DESeq (version 2.14) to determine the DEGs in each sample (FDR < 0.001), fold change ≥ 2). Ultimately, 2,926 DEGs were identified, among which 2,744 were upregulated and 182 were downregulated in the sample under blue light compared with that under red light (Fig 2C; S3 Table). Approximately 893 genes were upregulated by ≥ 2-fold, and 30 genes were downregulated by ≥ 2-fold.

### GO and KEGG pathways

**Fig 3:**
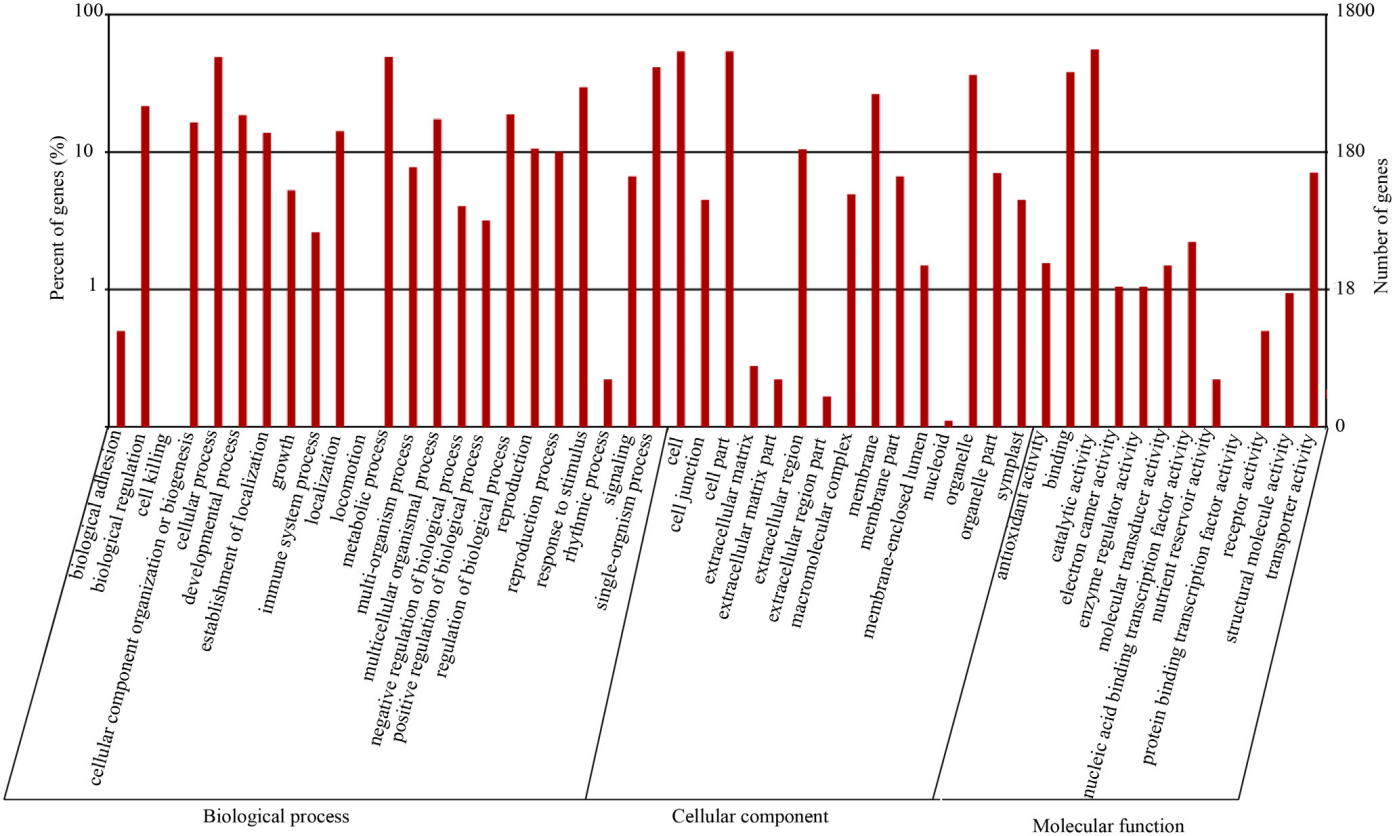
GO classification of DEGs.

GO terms were assigned to the mapped genes, and enrichment analysis of these terms showed that “metabolic process”, “cell and cell part”, and “catalytic activity” were predominant in the cellular component, molecular function, and biological process categories, respectively (Fig 3). Morphogenesis (“anatomical structure morphogenesis”, “post-embryonic morphogenesis”, “post-embryonic organ morphogenesis”, and “floral organ morphogenesis”), signaling pathway (“transmembrane receptor protein tyrosine kinase signaling pathway”, “enzyme-linked receptor protein signaling pathway”, and “cell surface receptor signaling pathway”), and growth regulation (“regulation of cell size”, “cell division”, “regulation of meristem growth”, and “regulation of meristem development”) were also significantly enriched in the biological process category (S4 Table).

**Fig 4:**
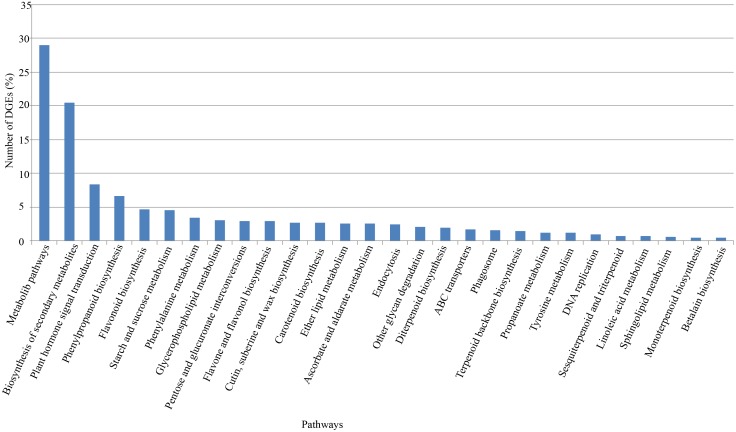
DEG pathways (Q-values of less than or equal to 0.05).

To identify the biological pathways that are active in Norway spruce under red light and blue light, we mapped the 2926 DEGs to the reference canonical pathways in the KEGG database. In total, 1669 DEGs were assigned to 111 KEGG pathways. Some of the genes assigned to primary metabolic process terms were associated with the following KEGG pathways: metabolic pathways (29%), biosynthesis of secondary metabolites (20.49%), plant hormone signal transduction (8.39%), phenylpropanoid biosynthesis (6.65%), flavonoid biosynthesis (4.67%), and starch and sucrose metabolism (4.49%) (Fig 4; S4 Table). These annotations increase the current understanding of the regulatory functions of light quality on specific processes, functions, and pathways in conifers.

**Fig 5:**
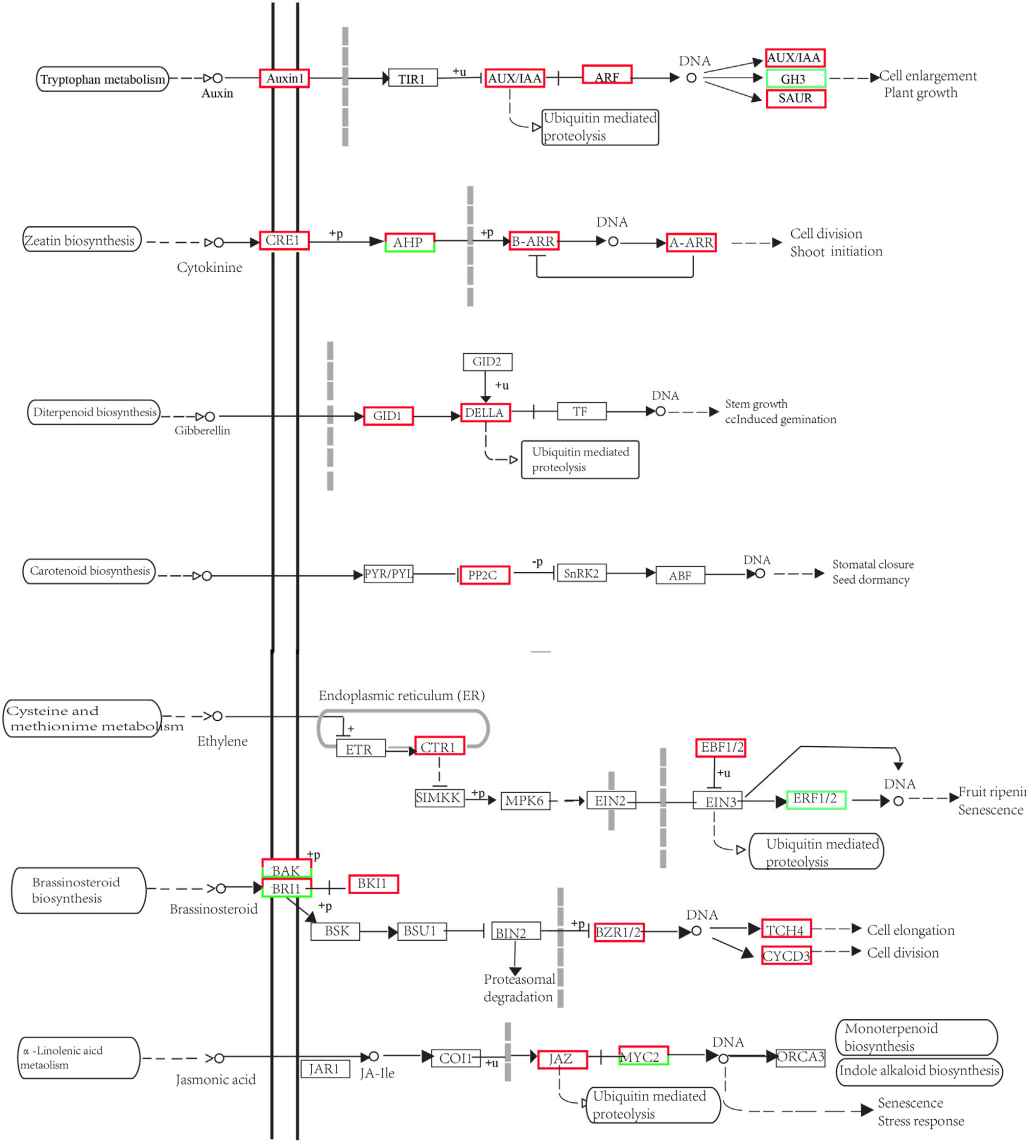
Heatmap of genes from metabolic pathways and biosynthesis of secondary metabolites.

There were 484 and 342 genes assigned to metabolic pathways and biosynthesis of secondary metabolites, respectively. Of these, only 33 and 36 genes were downregulated under blue light. Their heatmaps according to gene expression level [log_10_(RPKM)] are displayed in Fig 5. The KEGG pathway maps contained 39 metabolic pathway maps and 25 biosynthesis of secondary metabolites pathway maps. The significantly prominent pathways in the present study were phenylpropanoid biosynthesis, flavonoid biosynthesis, flavone and flavonol biosynthesis, starch and sucrose metabolism, phenylalanine metabolism and glycerophospholipid metabolism, which are presented in Table 2. The pathways revealed extensive blue-light-associated upregulation genes compared with those under red light treatment, specifically, 77.55%~98.21% upregulated (Fig 5, Table 2).

**Table 2:** Significantly enriched pathways of differentially expression unigenes.

Pathway category	Pathway map No.	Unigenes No.	%	Q-value	The upregulation genes under blue light compared to red light (%)
**phenylpropanoid biosynthesis**	map00940	111	6.65	8.92e-7	101 (90.99)
**flavonoid biosynthesis**	map00941	78	4.67	2.52e-3	62 (79.49)
**flavone and flavonol biosynthesis**	map00944	49	2.94	1.75e-3	38 (77.55)
**starch and sucrose metabolism**	map00500	75	4.49	5.86e-6	73 (97.33)
**phenylalanine metabolism**	map00360	56	3.36	5.63e-5	55 (98.21)
**glycerophospholipid metabolism**	map00564	50	3	1.21e-2	49 (98)

**Fig 6:**
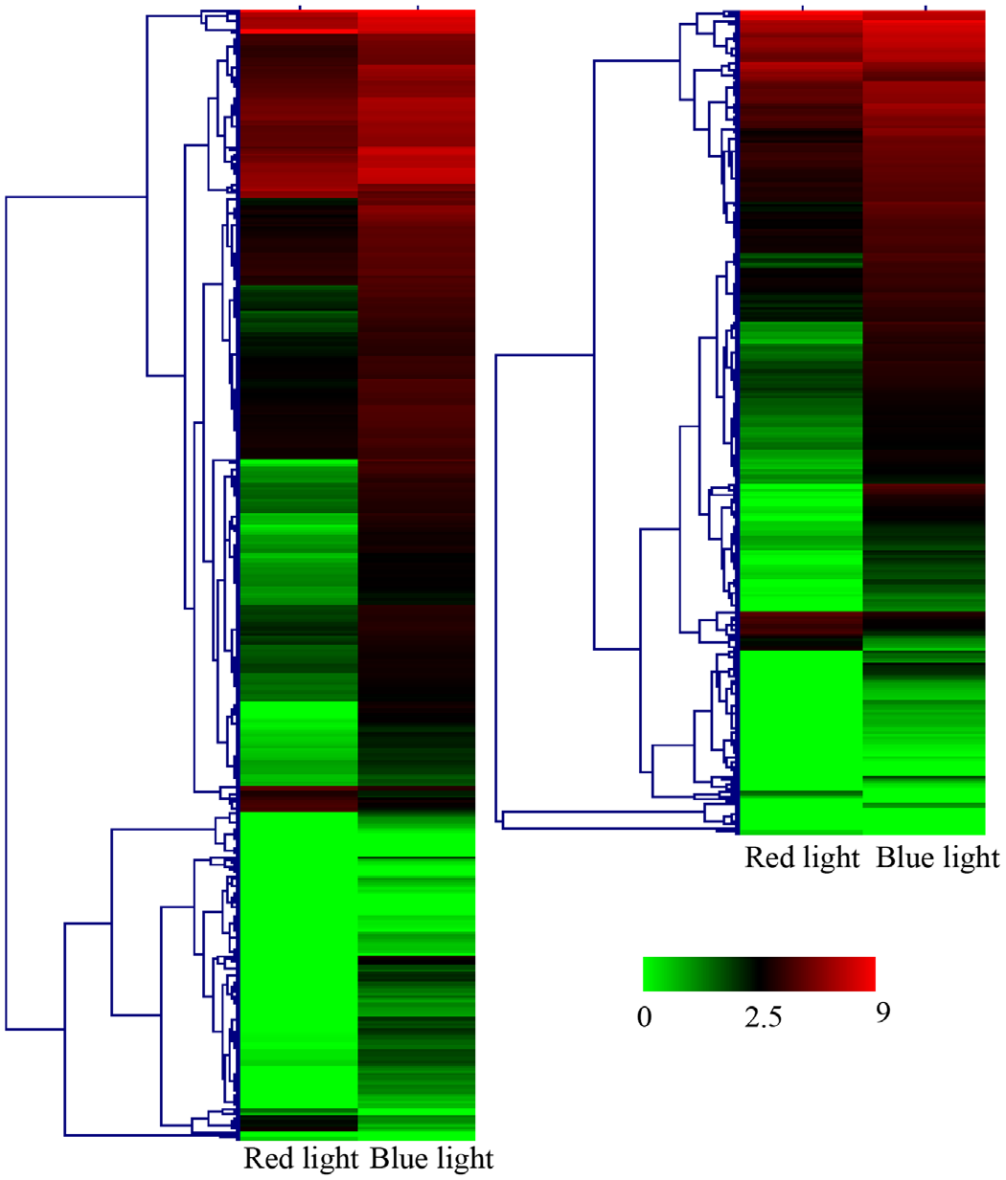
Plant hormone signal transduction.

By examining plant hormone signal transduction (Fig 6; S4 Table), we found that the *AUXIN-RESISTANT1* (*AUX1*), *AUX/IAA*, auxin-inducible genes, *auxin response factor* (*ARF*) and *small auxin-up RNA* (*SAUR*) genes were also upregulated under blue light, whereas the *GH3* gene was downregulated (Fig 6). *GRAS* genes (S2 Table), *GIBBERELLIN INSENSITIVE DWARF1* (*GID1*) and *DELLA* genes were upregulated under blue light. *CRE1*, *B-ARR*, *A-ARR* and *AHP3* (S2 Table) genes were also upregulated under blue light, whereas *AHP1* and *AHP5* (S2 Table) genes were downregulated. Only the *protein phosphatase 2C* (*PP2C*) gene, which is involved in ABA signal transduction, was upregulated in the samples grown under red light (Fig 6). In the pathway of ethylene (ET) signal transduction, *CTR1* and *EBF1/2* were upregulated under blue light, whereas *ERF1/2* was downregulated. In the pathway of brassinosteroid (BR) signal transduction, *BKI1*, *BZR1/2*, *TCH4* and *CYCD3* were upregulated under blue light. Twenty *BAK1* genes were upregulated and 6 genes were downregulated in the samples grown under blue light; 16 *BRI1* genes were upregulated and 4 genes were downregulated in the samples grown under blue light. In the jasmonic acid (JA) signal transduction pathway, *JAZ* was upregulated, whereas 13 *MYC2* genes were upregulated and 1 gene was downregulated under blue light.

Several DEG-encoding enzymes involved in the circadian rhythm in plants, plant hormone signal transduction, carotenoid biosynthesis, carbon fixation in photosynthetic organisms, oxidative phosphorylation, and the pentose phosphate pathway were identified (Table 3). With regard to the circadian rhythm in plants, PIF3 was upregulated by blue light, whereas constans (CO), chalcone synthase (CHS), and amyloid precursor proteins (APRs) 3, 5, and 7 were upregulated by red light. For plant hormone signal transduction, AUX1, AUX1/IAA, ARF, SAUR, GID1, and DELLA were upregulated by blue light, whereas GH3 (Table 3) and CONSTANS-LIKE 7 (COL7; MA_91504g0010, S2 Table) were upregulated by red light. Enzymes involved in carotenoid biosynthesis, carbon fixation in photosynthetic organisms, oxidative phosphorylation, and the pentose phosphate pathway were upregulated by blue light.

**Table 3:** KEGG pathway enzymes encoded by a portion of the DEGs.

KEGG pathway	EC number	Enzyme	The genes expression under blue light compared to red light
**Circadian rhythm-plant**	PIF3	phytochrome-interacting factor 3	upregulation
**Circadian rhythm-plant**	CO		both
**Circadian rhythm-plant**	CHS		downregulation
**Circadian rhythm-plant**	APR3, 5, 7		downregulation
**Plant hormone signal transduction**	AUX1, AUX1/IAA, ARF, SAUR		upregulation
**Plant hormone signal transduction**	GH3		downregulation
**Plant hormone signal transduction**	GID1 DELLA		upregulation
**Carotenoid biosynthesis**	EC 1.3.5.6	zeta-carotene desaturase	both
**Carotenoid biosynthesis**	EC 1.14.13.90	zeaxanthin epoxidase	upregulation
**Carotenoid biosynthesis**	EC 1.13.11.51	9-cis-epoxycarotenoid dioxygenase	upregulation
**Carotenoid biosynthesis**	EC 1.14.13.93	(+)-abscisic acid 8'-hydroxylase	upregulation
**Carotenoid biosynthesis**	EC 1.1.1288		both
**Carbon fixation in photosynthetic organisms**	EC 4.1.1.49	phosphoenolpyruvate carboxykinase (ATP)	upregulation
**Carbon fixation in photosynthetic organisms**	EC 1.2.1.13	glyceraldehyde-3-phosphate dehydrogenase (NADP+) (phosphorylating)	upregulation
**Oxidative phosphorylation**	EC 1.9.3.1	cytochrome c oxidase cbb3-type subunit I	upregulation
**Oxidative phosphorylation**	EC 3.6.3.14	F-type H+-transporting ATPase subunit alpha	upregulation
**Oxidative phosphorylation**	EC 3.6.3.10	H+/K+-exchanging ATPase	upregulation
**Oxidative phosphorylation**	COX6B	cytochrome c oxidase subunit 6b	upregulation
**Oxidative phosphorylation**	F-type	F-type ATPase	upregulation
**Oxidative phosphorylation**	ATPase (Eukaryotes)	F-type ATPase, prokaryotes and chloroplasts	upregulation
**Oxidative phosphorylation**	g	Glycolysis / Gluconeogenesis	upregulation
**Pentose phosphate pathway**	EC 2.7.1.15	inositol-polyphosphate multikinase	upregulation

### qRT-PCR analysis

**Fig 7:**
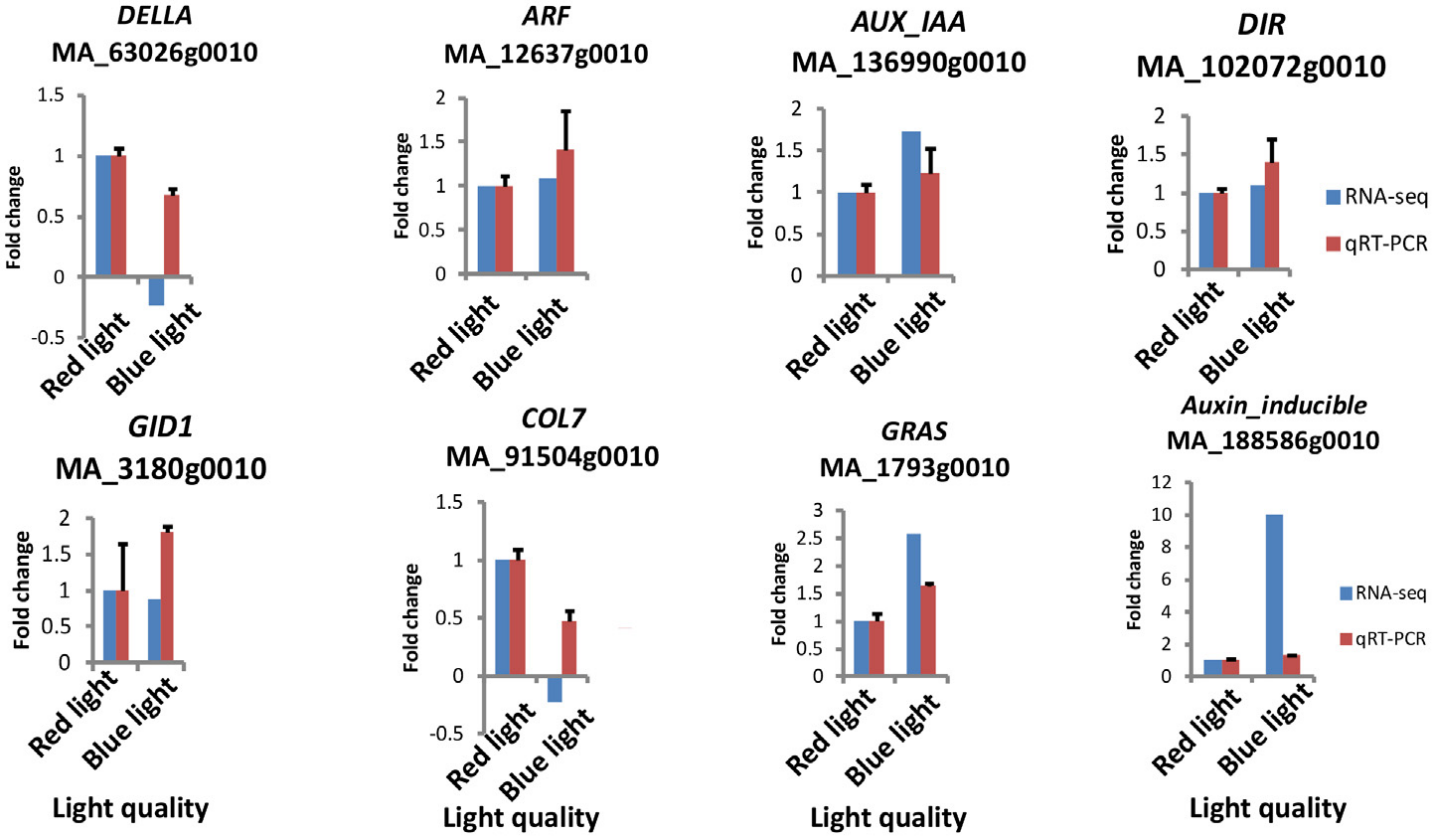
Validation of RNA-seq data by qRT-PCR.

Total RNA from the same two samples used for RNA-seq analysis was used as a template for qRT-PCR (Fig 7). For the majority of genes, the transcript fold changes determined by qRT-PCR were similar to those estimated from the RNA-seq data, supporting the RNA-seq results for *P*. *abies*.

## Discussion

The stem increment, ground diameter, leaf area, and leaf dry weight parameters of Norway spruce illuminated by red light were all higher compared with the samples illuminated by blue light, and these findings were coupled with a significantly higher GAs level and a lower IAA level. These changes may be related to DEG gene expression mainly associated with plant hormone signal transduction, metabolic pathways and biosynthesis of secondary metabolites. The DEG classifications revealed extensive blue-light-associated upregulation in Norway spruce.

Light-induced developmental and metabolic patterns in green plants are thought to be mediated primarily by changes in the expression of light-regulated genes [31], including those encoding photosynthetic components and enzymes [9]. In the present study, several genes involved in the response to red light or blue light were found to be differentially expressed; for example, MA_16729g0010, MA_41041g0010, and MA_10432538g0010 were upregulated under red light (S3 Table). Carotenoids absorb mainly blue-violet wavelengths and were upregulated under blue light (Table 2). These results show that blue/red light modulates plant growth and development by altering the expression of corresponding genes. Indeed, light quality influences plant growth by regulating specific photoreceptors [3, 32, 33], and photoreceptor gene expression was found to be affected by light quality in our study. However, significant differential expression between the two light qualities was not found, which is in accordance with previous reports of *Arabidopsis* seedlings [31] and *Saccharina japonica* (Phaeophyceae) [9]. The gene expression profiles of *Arabidopsis* seedlings grown under white light, red light, and blue light are very similar for most genes [31], and a large proportion of DEGs identified in *S*. *japonica* under blue light are also induced by red light [9]. These results indicate that light-regulated gene expression in Norway spruce is not a unique response to blue light or red light and that different light qualities are transduced to regulate the same metabolic patterns. Cryptochromes are both blue and red light receptors, suggesting that plant photoreceptors cooperate to control development and physiology [7]. In *Physcomitrella patens*, phototropins not only mediate blue light-induced chloroplast movement but also exhibit a function in chloroplast movement in response to red light, which it does not absorb [34].

GAs play a central role in promoting stem growth. GAs promote skotomorphogenesis and repress photomorphogenesis in contrast with light signals [17], accelerating stem elongation [35]. *Arabidopsis thaliana* mutants lacking endogenous GAs have shorter stems and smaller leaves [36]. In the present study, the GA concentrations were significantly increased under red light compared with blue light (Fig 1H), which might have been the reason for the greater height increase of the plants grown under red light in this study. In addition, the *GID1*, *DELLA*, and *GRAS* genes were upregulated under blue light (Fig 6, S2 Table). The GA-GID1 (GA receptor) complex can trigger the rapid degradation of DELLA proteins [37], a subfamily of *GRAS* genes belonging to a plant-specific transcription factor family, which includes *GIBBERELLIC ACID INSENSITIVE* (*GAI*), *REPRESSOR OF GAI* (*RGA*) and *SCARECROW* (*SCR*) [38]. As transcription factors, DELLA proteins in the nucleus play an important role in regulating sensitivity to GAs because they are involved in negative GA signaling [39]. Poplar has a decreased sensitivity to GAs because the levels of DELLA inhibitors (coding GA-INSENSITIVE) in apical buds increase rapidly when they are transferred to short-day conditions [40]. Olsen (2010) has proposed that the expression of *PIFs* increases under short-day conditions, which may stimulate the expression of *DELLA* inhibitors, leading to decreases in GA sensitivity and bud set [4]. PIFs are important factors linking light and plant hormone signaling and regulating seedling photomorphogenesis [41], and they can activate *GAI* and *RGA* expression [39]. *PIF3* and *DELLA* inhibitors were found to be upregulated by blue light in this study (Table 2), which is similar to a study of poplar indicating that *PIF3-LIKE1* and *PIF4* transcription increases following transfer to short-day conditions [42]. These results demonstrate that the mechanisms by which GAs control growth in Norway spruce (Fig 6) may involve the GA-GID1-DELLA signaling module of angiosperms [37], which is also in accordance with a model proposed by Olsen in which GAs control growth [4].

In addition to GAs, auxin has been suggested to be involved in growth cessation, cold acclimation, and dormancy induction [43]. Auxin also plays an important role in photomorphogenesis [44, 45]. Light signaling and the auxin pathway have been clearly demonstrated to be intertwined, and a series of AUX/IAA proteins are phosphorylated by phytochrome A [46]. In the present study, the IAA levels were significantly increased in the plants illuminated under blue light compared with those illuminated under red light (Fig 1G). In addition, the *AUX/IAA*, auxin-inducible, and early auxin-responsive genes (*ARF* and *SAUR*) were upregulated under blue light (S3 Table, Table 2, Fig 5). These results indicate that blue light promoted and red light suppressed auxin metabolism by regulating the expression of related genes. Reddy and Finlayson have also demonstrated that the red light receptor phytochrome B promotes branching in *Arabidopsis* by suppressing auxin signaling [23]. We found that *COL7* was upregulated under red light (S4 Table). COL7 is a critical factor linking photoreceptor and auxin levels and enhances the branching number and downregulates the IAA level under high red:far-red light [22]. COL7 also promotes the mRNA expression of *SUPERROOT 2* (*SUR2*), which suppresses auxin biosynthesis, in association with photo-excited phyB [22].

The roles of ABA and GAs in plant growth and development are antagonistic. ABA represses growth in contrast with GAs. Low red:far-red light quality suppresses bud outgrowth but not that of the topmost bud, which is notably associated with ABA, as shown by DEG analysis [47]. By contrast, the ABA level was not significantly affected by the two light qualities assessed in this study. In ABA signal transduction, only *PP2C* genes, which have been demonstrated to function as negative regulators of ABA signaling [48], were upregulated in the samples grown under blue light (Fig 6). Cytokinins are well known to control cell division in plant growth and development [49], promoting axillary bud outgrowth [50]. With regard to cytokinin signal transduction, *AHP3* was found to be upregulated under blue light; however, *AHP1* and *AHP5* were downregulated (Fig 6), which might have caused the lack of a significant difference in the ZR level between the two light qualities. It has been demonstrated that the five *Arabidopsis AHP* genes are ubiquitously expressed and unaffected by cytokinin [51]. *AHP1*, *AHP2* and *AHP5* overexpression has been found to have no effect on cytokinin primary response gene expression [52]. In addition to IAA, GAs, cytokinins and ABA, the signal transduction pathways of other hormones (ET, BR, JA) were also influenced by red light and blue light (Fig 6). ET, BR and JA all play an important role in the regulation of hypocotyl elongation of *Arabidopsis* seedlings in response to light [53–55]. Ethylene suppress hypocotyl elongation in darkness while promoting it in light [53]. Serine/threonine-protein kinase (*CTR1)* and *EBF1/2*, which were repressors in the ethylene signaling pathway, were all upregulated under blue light, whereas an ethylene response transcription factor (*ERF1/2*) was downregulated under blue light (Fig 6). *ERF1* mediates an ethylene activated growth-inhibition pathway that operates effectively in the dark and minimally under strong light conditions in *Arabidopsis* [53]. *JAZ* and most of the *MYC2* genes were upregulated under blue light in the pathway of JA signal transduction (Fig 6). JAZs are repressors of transcription factors that are positive regulators of JA responses. However, *MYC2* acts as a repressor of blue light-mediated photomorphogenic growth in *Arabidopsis* [54]. Both auxin and brassinosteroid (BR) play an important role in regulating the enhanced hypocotyl elongation of *Arabidopsis* seedlings in response to blue light depletion [55]. *BRI1* is a receptor-like kinase in the BR signaling pathway, which then triggers downstream signaling components. In the present study, most of the *BRI1* genes were upregulated in the samples under blue light (Fig 6). BR-regulated plant growth usually depends on an intact auxin signaling pathway [56].

The light spectrum is an important environmental factor that regulates plant growth and development and also influences the secondary metabolism, which acts as de defense compounds [57]. In the present study, blue light promoted phenylpropanoid biosynthesis and phenylalanine metabolism with 90.99% and 98.21% of the genes upregulated, respectively (Table 2). Phenylalanine ammonia-lyase (PAL) is a key enzyme in the phenylpropanoid pathway. The *PAL* gene is upregulated under blue light (MA_44561g0010, S2 Table), which is consistent with the findings in lettuce (*Lactuca sativa* L.) [58]. Lignins and flavonols are derived from multiple branches of the phenylpropanoid biosynthesis pathways. In the present study, blue light promoted dirigent gene families, which were related to lignin biosynthesis and conifer defense [59] (S3 Table, Fig 7). Chalcone synthase (CHS), the first enzyme in flavonoid biosynthesis, is only expressed under blue light (MA_84838g0010, S2 Table). In addition, more than 77% genes are upregulated under blue light in flavonoid biosynthesis and flavone and flavonol biosynthesis (Table 2). Blue light facilitates the accumulation of flavonoids in *Arabidopsis* ([60]), lettuce [58] and *Saccharina japonica* (Laminariales, Phaeophyceae) [25], which might reduce the primary metabolites for plant growth. There are interaction effects between plant hormones and second metabolism [61]. Flavonoids act as negative regulators of auxin transport *in vivo* in *Arabidopsis* [26]. Auxin transport is elevated in the absence of endogenous flavonoids [26]. However, blue light promoted flavonoid biosynthesis of clones with a higher IAA level in the present study, which may be correlation with other hormones. Ethylene may reportedly be involved in regulating light-induced phenylpropanoid accumulation in the tea plant [62].

The qRT-PCR quantification of transcript abundance for eight hormone- and secondary-metabolism-associated genes in the samples under blue and red light for 90 days were similar to those estimated from the RNA-seq, which indicated that the high reliability results had an certain reference value. The time course of apical bud formation in white spruce (*Picea glauca*) [35] showed that endogenous hormone content and related gene expression level were stable during 70 days under short-day treatment. Possibly, the gene expression level-associated hormone and second metabolism of Norway spruce under long-day treatment for 90 days were also stable. However, this finding requires further verification. A time-course expression analysis of candidate genes involved in plant hormone signal transduction and secondary metabolism under different light treatment should be conducted in the future to determine whether phytohormones are directly or indirectly affected by light quality, as well as their effects on other pathways.

In conclusion, light quality regulates Norway spruce seedling growth and development by mainly affecting the metabolic pathway, biosynthesis of secondary metabolites and plant hormone signal transduction by altering the expression of corresponding genes identified by RNA-seq. Blue light promotes IAA accumulation and second metabolism biosynthesis, and red light promotes GA accumulation in Norway spruce.

## Supporting Information

S1 Table Primer sequences used in qRT-PCR. (XLS)

S2 Table 21,923 unique genes expressed between the two libraries. (XLS)

S3 Table 2,926 DEGs identified between the two libraries. (XLS)

S4 Table GO classification (P-values of less than 0.05) and KEGG pathway (Q-values of less than or equal to 0.05) assignments of DEGs. (XLS)
